# The synergy of depression and flourishing/languishing on suicidal thoughts and behaviors: Findings from a national sample of emerging adult students in higher education in the United States

**DOI:** 10.1371/journal.pone.0309020

**Published:** 2024-08-27

**Authors:** Hans Oh, Louis Jacob, Nirit Soffer-Dudek, Jae Il Shin, Lee Smith, Megan Besecker, Edouard Leaune, Trevor A. Pickering

**Affiliations:** 1 University of Southern California, Los Angeles, CA, United States of America; 2 Research and Development Unit, Parc Sanitari Sant Joan de Déu, CIBERSAM, ISCIII, Sant Boi de Llobregat, Barcelona, Spain; 3 Department of Physical Medicine and Rehabilitation, AP-HP, Lariboisière-Fernand Widal Hospital, Université Paris Cité, Paris, France; 4 Inserm U1153, Epidemiology of Ageing and Neurodegenerative Diseases (EpiAgeing), Université Paris Cité, Paris, France; 5 Department of Psychology, Ben-Gurion University of the Negev, Beersheba, Israel; 6 Department of Pediatrics, Yonsei University College of Medicine, Seoul, South Korea; 7 Severance Underwood Meta-Research Center, Institute of Convergence Science, Yonsei University, Seoul, Republic of Korea; 8 Centre for Health, Performance and Wellbeing, Anglia Ruskin University, Cambridge, United Kingdom; 9 Center for Suicide Prevention, Centre Hospitalier Le Vinatier, Bron, France; 10 RESearch on HealthcAre PErformance (RESHAPE), INSERM U1290, Université Claude Bernard Lyon 1, Lyon, France; Kitami Institute of Technology, JAPAN

## Abstract

**Background:**

Suicide is a leading cause of death among students in higher education, driven in large part by mental illness, but also mental wellness. Relatively few studies have examined the extent to which depression and flourishing/languishing interact in relation to suicidal thoughts and behaviors.

**Methods:**

We analyzed data from the Healthy Minds Study (2020–2021; emerging adult students aged 18–29; N = 101,435), and calculated interaction contrast ratios to estimate the interaction between depression and flourishing/languishing on suicidal thoughts and behaviors, using an additive scale, adjusting for age, gender, race/ethnicity, and food insecurity.

**Results:**

When compared with students who were flourishing without depression, the students who were languishing without depression, and the students who were depressed but still flourishing had significantly greater odds of suicidal thoughts and behaviors. However, students who were depressed and languishing had the greatest odds, exceeding the sum of the individual effects.

**Conclusion:**

The interaction of depression and flourishing/languishing produced a synergy that increased odds of suicidal thoughts and behaviors. Flourishing interventions may prove to be an effective strategy for universal suicide prevention.

## Introduction

Suicide is the second leading cause of death among college students in the United States, with approximately 1,100 suicides on campuses per year [1], calling for interventions informed by a comprehensive understanding of risk and protective factors [2, 3]. A substantial body of literature links depression to suicidal thoughts and behaviors (STB; [4]), though depression alone does not account for the entirety of suicide risk, since people can die by suicide without having any psychiatric diagnosis [5]. While mental illnesses like depression are key drivers of STB, mental wellness is also important, yet far fewer studies have examined flourishing with respect to STB.

Fredrickson and Losada [6] described flourishing as “[living] within an optimal range of human functioning, one that connotes goodness, generativity, growth, and resilience.” To be clear, flourishing is not merely the absence of psychological symptoms, deficits, or disorders; it refers to the presence of positive things such as having purpose in life, having meaningful relationships, and feeling respected [7–9]. When people are not flourishing, they experience what is known as *languishing*. It is estimated that in 2020–2021, only around 1-in-3 students in higher education were flourishing during the first year of the COVID-19 pandemic [10]. The prevalence of languishing is an concern in its own right, but adding to this concern are its associations with a range of mental and physical health problems [11].

### Study aims

Studies are also showing flourishing is negatively related to STB [12–14], though more research is needed to understand flourishing vis-à-vis depression, and their potential interactions. Since depression and flourishing are conceptualized along two different continua [8], it is possible for people to (a) have depression while flourishing; (b) languish without any depression; and (c) have depression while languishing. However, depression and flourishing/languishing are rarely examined together in relation to STB. In this study, we analyzed data from a large sample of students in higher education during the COVID-19 pandemic, when depression, STB, and languishing increased [15–17]. We examined whether depression and flourishing/languishing interacted synergistically to increase odds of STB.

## Methods

### Sample

We analyzed data from the Healthy Minds Study (HMS; 2020–2021), a non-probability online survey administered to students in higher education in the United States. Between September 2020 through June 2021, the survey was administered at 140 public and private institutions of higher learning. The total response rate was 14%. To focus on emerging adults, we restricted the sample by to individuals aged 18–29, and we used complete-case analysis, resulting in weighted samples ranging from 101,435 (for the suicide attempt model) to 101,581 (for the suicidal ideation model). The HMS was approved by the Institutional Review Board Advarra, and the Institutional Review Board at the University of Southern California. The HMS data are available upon request at: https://healthymindsnetwork.org/hms/.

### Measures

#### Past year suicidal thoughts and behaviors (dependent variables)

STB were measured using binary variables that asked about: ideation (*In the past year*, *did you ever seriously think about attempting suicide*?), plans (*In the past year*, *did you make a plan for attempting suicide*?), and attempts (*In the past year*, *did you attempt suicide*?). The questions about suicide plan and attempt were only given if the respondent answered affirmatively to ideation.

#### Depression

Depression was measured using the validated and widely used Patient Health Questionnaire– 9 (PHQ-9; [18]). The PHQ-9 contains elicits the frequency of nine depressive symptoms over the past two weeks. Response choices ranged from ‘not at all’ to ‘nearly every day’. Depression items were summed into a scale [0–27] and then dichotomized to reflect moderately severe to severe depression (>15).

#### Flourishing/languishing

We assessed flourishing/languishing using a validated scale [7, 19], which elicits the respondent’s level of agreement to eight statements, such as *I lead a purposeful and meaningful life* and *I actively contribute to the happiness and wellbeing of others*. Respondents could answer: *strongly disagree*, *disagree*, *mixed/neither agree nor disagree*, *slightly agree*, *agree*, *strongly agree*. We summed the items into a scale [8–56], with higher scores representing greater levels of flourishing. The flourishing scale was dichotomized such that a score of 47 or lower was considered languishing, and scores above were considered flourishing [20].

#### Sociodemographic covariates

Respondents self-reported sociodemographic characteristics, including age (continuous), gender (cisgender man, cisgender woman, and transgender/non-binary/self-report), race/ethnicity (White, Black/African American, Asian American or Native Hawaiian/Pacific Islander, Hispanic/Latinx, Multiracial, and Other [which includes Middle Eastern/Arab/Arab American, American Indian/Alaskan Native]). We also adjusted for food insecurity, which is prevalent among students in higher education and a useful metric for socioeconomic status in this population, as it can take into account financial support from family [21–23]. Food insecurity was measured using two items where respondents were asked the extent to which they agreed with the following statements: (1) *Within the past 12 months I was worried whether our food would run out before we got money to buy more*; and (2) *Within the past 12 months*, *the food I bought just didn’t last and I didn’t have money to get more*. Respondents could answer: *never true*, *sometimes true*, *often true*. Individuals were considered food insecure if they answered *sometimes true* or *often true* to either question [24, 25].

### Analysis

We calculated the prevalence of depression, flourishing/languishing, their co-occurrence, and STB. We tested for interactions using an additive scale and depicted the synergy between depression and flourishing/languishing on STB (suicidal ideation, suicide plan, and suicide attempt) by creating the following categorical variable: (1) flourishing and no depression (reference group); (2) depressed but flourishing; (3) languishing but not depressed; and (4) both depressed and languishing. We adjusted for age, gender, race/ethnicity, and food insecurity. We calculated the interaction contrast ratios (ICRs), which allows use of odds ratios derived from logistic models to estimate the relative excess risk resulting from the synergy of combined effects. Confidence intervals and p-values for ICRs were generated using the nlcom command in Stata SE 15. We used sample probability weights to adjust for non-response using administrative available data on full student populations at each institution. We present all results as odds ratios with 95% confidence intervals, with statistical significance set at p-value<0.050.

## Results

Sample characteristics for the HMS data have been described in detail elsewhere [10, 26] and are presented in **Table 1**. The analytic sample for this current study was majority White (~61%) and cisgender women (~57%). The mean age was 21 (95% CI: 21.0–21.3). Around 5.8% of students (n = 5804) reported depression but were flourishing, 27.4% reported languishing but without depression (n = 28,286), and 36% reported both depression and languishing (n = 38,034). Over the past 12 months, about 14.2% reported suicidal ideation (n = 13,545), 5.9% reported a suicide plan (n = 5403), and 1.5% reported a suicide attempt (n = 1233).

**Table 1:** Sample characteristics.

	No 12-month suicide attempt (n = 100,202)	12-month Suicide attempt (n = 1,233)	Total (N = 101435)	P-value
**Suicidal ideation**				<0.001
No	87880 (85.82%)	0 (0.00%)	87880 (85.82%)	
Yes	12313 (12.73%)	1232 (1.45%)	13545 (14.18%)	
**Suicide plan**				<0.001
No	95815 (93.87%)	207 (0.23%)	96022 (94.10%)	
Yes	4378 (4.68%)	1025 (1.22%)	5403 (5.90%)	
**Depression, Flourishing/Languishing**				<0.001
Flourishing and no depression	29254 (29.92%)	56 (0.05%)	29310 (29.97%)	
Depressed but flourishing	5745 (5.73%)	58 (0.10%)	5803 (5.83%)	
Languishing but not depressed	28143 (27.27%)	141 (0.16%)	28284 (27.43%)	
Depressed and languishing	37051 (35.63%)	977 (1.14%)	38028 (36.77%)	
**Age**	21.18 (21.02–21.33)	20.48 (20.28–20.69)	21.17 (21.01–21.32)	<0.001
**Gender**				<0.001
Man	27554 (39.03%)	248 (0.45%)	27802 (39.47%)	
Woman	69199 (56.22%)	839 (0.86%)	70038 (57.08%)	
Trans, nonbinary, queer	3313 (3.18%)	143 (0.15%)	3456 (3.32%)	
**Race/ Ethnicity**				<0.001
White	61578 (59.82%)	648 (0.73%)	62226 (60.55%)	
Asian Pacific Islander	11985 (9.19%)	150 (0.15%)	12135 (9.34%)	
Black	8332 (10.51%)	162 (0.23%)	8494 (10.74%)	
Hispanic	6797 (7.80%)	92 (0.12%)	6889 (7.92%)	
Two or More	9501 (9.42%)	156 (0.19%)	9657 (9.61%)	
Other	1626 (1.39%)	17 (0.02%)	1643 (1.41%)	
Missing/unknown	374 (0.42%)	7 (0.01%)	381 (0.43%)	
**Food insecurity**				<0.001
No	73629 (68.73%)	526 (0.59%)	74155 (69.32%)	
Yes	26564 (29.83%)	706 (0.86%)	27270 (30.68%)	

P-values by t-test for continuous variables and Chi2 test for binary/categorical variables.

**Fig 1:**
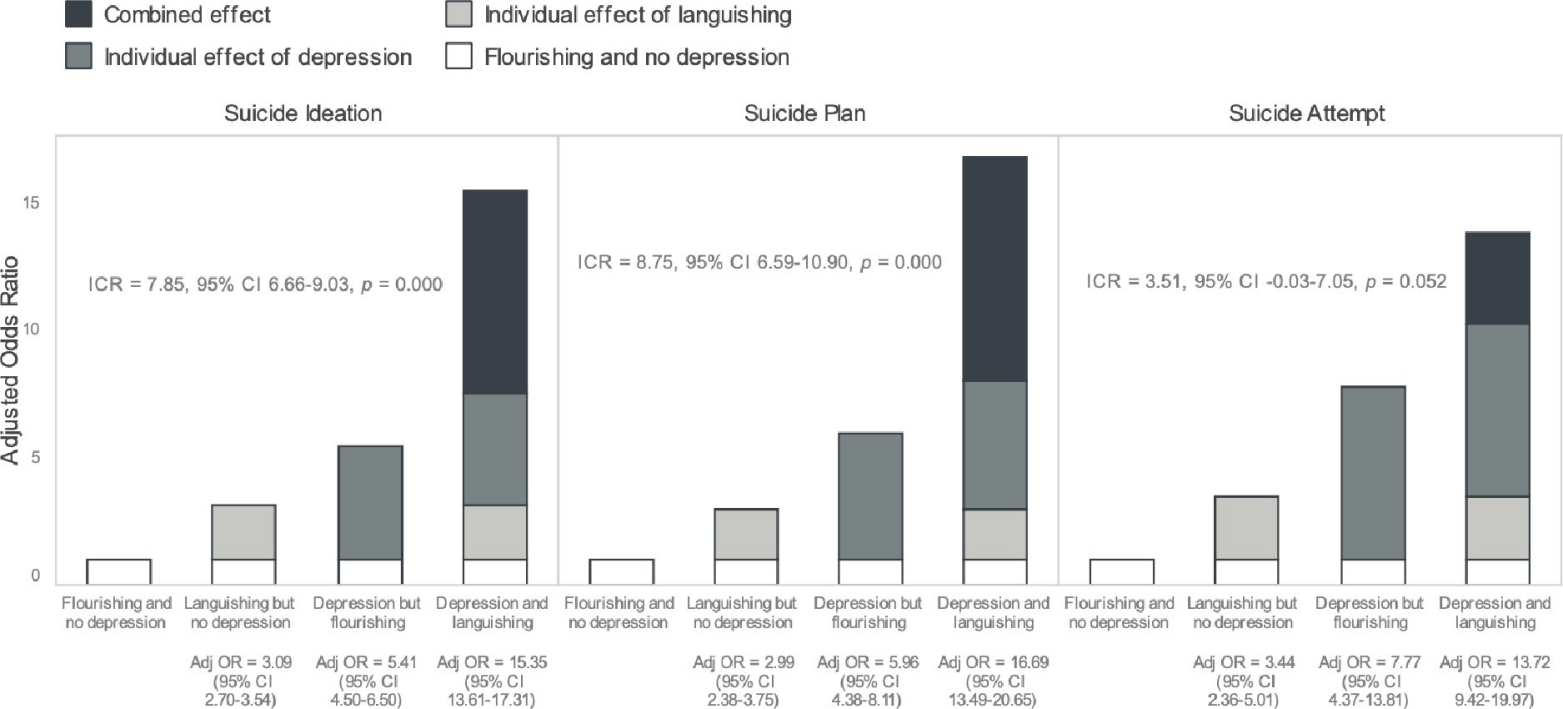
Synergistic effects of languishing and depression on suicidal thoughts and behaviors.

**Fig 1** shows the synergy of depression and languishing on odds of STB on an additive scale. When compared with flourishing without depression (reference group), languishing without depression was associated with a little over three-fold greater odds of suicidal ideation (aOR: 3.09; 95% CI: 2.70–3.54), and depression while flourishing was associated with over 5.4 times greater odds of suicidal ideation (aOR: 5.41; 95% CI: 4.50–6.50). Those who endorsed both depression and languishing had the greatest odds of suicidal ideation (aOR: 15.35; 95% CI: 13.61–17.31), exceeding the sum of the individual effects, specifically 7.85 higher odds of suicidal ideation than if there were no synergy between depression and languishing (ICR: 7.85, 95% CI: 6.66–9.03). This same pattern was evident for suicide plan and suicide attempt, though the ICR was smaller for these outcomes, and the ICR was only marginally significant (p = 0.052) for suicide attempt.

## Discussion

### Main findings

In this study, we treated flourishing/languishing as conceptually distinct from depression and found that they both contributed to STB among a diverse sample of emerging adult students in a higher education in the United States. We found that when compared with students who were flourishing without depression, students who were languishing but not depressed were more than three times as likely to have had suicidal ideation over the past year, while students who were depressed but flourishing had between five and six times as likely to have had suicidal ideation. Our findings point to the importance of having positive elements of life—such as having purpose, engaging fully in daily activities, feeling competent while performing important activities, and feeling like a good person [6, 19]. However, depression still had more potent effects on STB. Notably, we found that students who were both depressed and languishing were over 15 times as likely to have suicidal ideation, and this synergy was also evident for suicide plans and suicide attempts, though to a lesser degree for suicide attempts. These associations persisted even while adjusting for sociodemographic characteristics (age, gender) and socioeconomic status (food insecurity), which have been correlated with depression, flourishing, and STB in various populations [23, 27–29]. Our study corroborates prior studies linking flourishing to suicide risk, including one study of men with criminal records in China [13], and another study of adolescents in Spain [14]. To our knowledge, this was the first study to report a synergy between flourishing/languishing and depression in a large racially/ethnically diverse sample of students in higher education in the US.

### Limitations

Our findings should be interpreted bearing in mind several limitations. First, in terms of sampling, the response rate was 14%. This limitation is not uncommon for online convenience samples and panels [30, 31], and was mitigated somewhat through survey weights adjusting for nonresponse. However, sampling bias remains a concern. In terms of measurement, all measures were self-reported and responses may have been influenced by recall and social desirability biases. For example, respondents may have been reluctant to disclose STB even though the surveys were anonymous. In terms of study design, all data were cross-sectional and we could not infer causal direction. While languishing may lead to STB, it is conceivable that the causal direction can be reversed or bidirectional. One study found that flourishing may reduce the risk of incident mood disorders [32], and that changes in positive mental health predicted the prevalence and incidence of psychiatric disorders (including depressive episode) [9]. More longitudinal studies are needed to examine temporal order of events and their associations over time. In terms of generalizability, the survey was administered during the first year of the COVID-19 pandemic, when languishing was elevated for college students [10]. Thus, it is unknown whether the findings are necessarily generalizable to other (non-pandemic) time periods and to other populations outside of higher education.

### Implications

Findings suggest that flourishing may play an important buffering role in the association between depression and STB. One possibility is that flourishing can inform screening and prevention. Flourishing and languishing tend to be less stigmatizing to talk about and may serve as a softer point of entry into conversations about depression or STB. Moreover, individuals may not necessarily feel motivated to address depression through traditional therapies but may be more open to flourishing interventions. One literature review on flourishing interventions drew from expert feedback to arrive at a comprehensive 3-month group intervention (90 min each session, weekly). The intervention covered topics such as: mental and physical health, virtues and character strengths, love, gratitude, kindness, volunteering, happiness, social support, family, friends and community, forgiveness, compassion, resilience, spirituality, purpose and meaning of life, and imagining the ‘best possible future’ [33]. These intervention components can easily be incorporated into traditional depression treatment (e.g., using behavioral activation to volunteer for a meaningful cause).

In terms of scope, the benefit that flourishing interventions have over other traditional mental health treatments is that they can be implemented broadly [34]. The field of preventive medicine and suicide prevention often weighs the costs and benefits of using selective, indicated, and universal interventions, as administering an intervention to someone who does not need it can be a waste of resources and can be needlessly stigmatizing [35–37]. However, flourishing interventions tend not to carry the same stigma as mental health treatment and may therefore be more appropriate for universal prevention strategies, since flourishing is broadly beneficial regardless of whether the person ultimately develops depression or STB. That is, promoting flourishing may buffer the impact of depression or directly reduce STB, but could also in theory enhance academic performance, reduce loneliness, and improve overall quality of life, which are desirable outcomes irrespective of suicide risk. Therefore, flourishing interventions can be administered at counseling centers for indicated and selective interventions, but could also be normalized and promoted on campuses, available online, and advertised during student orientations, for everyone to utilize. Some universities offer courses on happiness and flourishing, which are popular and well-received [38].

## Conclusion

Our findings support the idea that flourishing/languishing and depression synergistically interact to increase odds of STB among emerging adults in higher education. Promoting flourishing is generally beneficial for almost everyone and serves as an ideal target for universal preventive interventions. Wide-scale interventions to cultivate flourishing may prove to be helpful for emerging adult students to reduce mental health problems, including STB, while increasing functioning and life satisfaction.
